# Ideal Cardiovascular Health Metrics Associated with Reductions in the Risk of Extracranial Carotid Artery Stenosis: a Population-based Cohort Study

**DOI:** 10.1038/s41598-018-29754-3

**Published:** 2018-08-16

**Authors:** Junyou Wang, Bo Shao, Da Lin, Xijun He, Yongqiang Zhang, Li Zhang, Tian Jiang, Jinzhong Xu, Youxin Wang, Jing Wu, Yong Zhou, Junzheng Chen, Lingfang Teng

**Affiliations:** 1 Department of Neurosurgery, The First People’s Hospital of Wenling, Wenling, 317500 China; 2 Department of Neurology, The First People’s Hospital of Wenling, Wenling, 317500 China; 3 Department of Central Lab, The First People’s Hospital of Wenling, Wenling, 317500 China; 4 Department of Clinical Pharmacy, The First People’s Hospital of Wenling, Wenling, 317500 China; 5 Beijing Key Laboratory of Clinical Epidemiology, School of Public Health, Capital Medical University, Beijing, 100069 China; 6 Beijing Recdata Technology Co., Ltd., Beijing, 100050 China; 7 Department of Cardiology, Beijing An Zhen Hospital, Capital Medical University. Beijing Institute of Heart, Lung and Blood Vascular Diseases, Beijing, 100029 China; 8 Department of General Surgery, The First People’s Hospital of Wenling, Wenling, 317500 China

## Abstract

The cardiovascular health (CVH) metrics are closely related to the risk of stroke. Extracranial carotid artery stenosis (ECAS) represents an important risk factor for ischemic stroke. The present study aims to explore the longitudinal effect of the baseline CVH metrics on the development of ECAS. Totally 5,440 participants were randomly enrolled in the Asymptomatic Polyvascular Abnormalities Community study from 2010 to 2011. Information regarding the seven CVH metrics was collected at baseline. ECAS was assessed by performing carotid duplex sonography at baseline (2010–2011) and during the follow-up (2012–2013). Finally 3,487 subjects were included, and 976 participants developed ECAS during the 2-year follow-up. The optimum CVH status was associated with a 42% (95% confidence interval: 0.40–0.85) decreased risk of the incidence of ECAS after adjusting for age, sex, weight, education, income, alcohol use, waist-hip ratio, triglycerides, low-density lipoprotein cholesterol, high-density lipoprotein cholesterol, uric acid, homocysteine, and C-reactive protein. Ideal physical activity, total cholesterol and fasting blood glucose were independent protective factors of ECAS. In this cohort study, the ideal baseline CVH status was negatively associated with the occurrence of ECAS during the follow-up. This study provides practical insight for further developing effective screening strategies or implementing the best medical treatment.

## Introduction

Stroke is the second leading global cause of death and accounts for 11.13% of all deaths worldwide, and 87% of stroke-related deaths are caused by ischemic stroke^1^. In China, data from the Global Burden of Diseases, Injuries, and Risk Factors Study 2010 (GBD 2010) indicated that stroke is the leading cause of death^2^. Extracranial carotid artery stenosis (ECAS), which is usually attributed to atherosclerosis, is a well-known risk factor for ischemic stroke^3^, leading to 20% of cerebral ischemia^4^. The etiological differentiation of ischemic stroke is classified into the following 5 subtypes: (1) large artery, (2) cardioembolic, (3) small artery, (4) miscellaneous, and (5) cryptogenic origin^5^. In America, the pathogenic distribution was approximately as follows: 16% large artery, 16% small artery, 29% cardioembolic, 3% miscellaneous, and 36% cryptogenic origin^6^. Generally, atherosclerosis is considered as a chronic systemic inflammatory disease that affects medium- and large-sized arteries^7^. The atherosclerotic extracranial carotid artery causes moderate to severe stenosis and usually remains silent until triggering acute cerebral ischemia via distal atheroembolization or, less commonly, arterial thrombosis^7^. Thus, effective prevention remains the best approach for reducing the stroke burden^4^.

To reduce cardiovascular mortality by 20% by 2020, the American Heart Association (AHA) proposed seven cardiovascular health (CVH) metrics in 2010^8^, including smoking status, body mass index (BMI), physical activity, diet, fasting plasma glucose (FBG), blood pressure (BP), and total cholesterol (TC). The AHA proposed a shift in emphasis from disease prevention to the prevention of the emergence of the risk factors, namely, primordial prevention^9^. Several studies have demonstrated that the ideal CVH is negatively correlated with coronary heart disease, stroke, atherosclerosis, vascular intima-media thickness, brachial-ankle pulse wave velocity, suboptimal health status, and cancer^10–15^. A previous cross-sectional study revealed a negative correlation between the ideal CVH metrics and the prevalence of ECAS in northern Chinese adults^16^. However, whether the ideal CVH metrics affect the incidence of ECAS in the general population remains unclear. In this longitudinal study, we aimed to investigate whether individual ideal CVH statuses influence the incidence of ECAS in the general population.

## Results

As shown in Table 1, 55.43% (1,933/3,487) of the study population were male, with a mean age of 53.94 ± 10.71 years, and the average summary CVH score was 8.66 ± 2.20 points. During the 2-year follow-up, 976 participants developed ECAS. The incidence of <50% stenosis and ≥50% stenosis was 27.22% (949/3,487) and 0.77% (27/3,487), respectively. The difference in the baseline characteristics is shown according to the CVH scores. The individuals in the inadequate group were more likely to be elderly, male, heavier in weight, less educated, high-income earners, and drinkers, and to have a higher waist-hip ratio, higher triglycerides (TG), higher low-density lipoprotein cholesterol (LDL-C), lower high-density lipoprotein cholesterol (HDL-C), higher uric acid (UA), higher homocysteine, and higher C-reactive protein (CRP).

**Table 1:** Baseline characteristic comparisons among the participant with the different CVH scores.

	Total (n = 3487)	Inadequate (0~4 points)	Average (5~9 points)	Optimum (10~14 points)	*P-value*
		n = 101	n = 2124	n = 1262	
Age, years	53.9 ± 10.7	55.2 ± 10.1	55.6 ± 10.8	51.0 ± 10.0	<0.001
Sex, %					<0.001
Female	1554 (44.6)	8 (8.0)	664 (31.3)	882 (69.9)	
Male	1993 (55.4)	93 (92.1)	1460 (68.7)	380 (30.1)	
Body weight, kg	69.0 ± 11.0	81.5 ± 10.9	71.7 ± 10.6	63.3 ± 9.0	<0.001
Education level, %					<0.001
Illiterate/primary school	343 (9.8)	11 (10.9)	242 (11.4)	90 (7.1)	
Middle school	1483 (42.5)	48 (47.5)	954 (44.9)	481 (38.1)	
High school or above	1660 (47.6)	42 (41.6)	927 (43.7)	691 (54.8)	
Income, ¥/month, %					0.007
<¥1000	729 (20.9)	12 (11.9)	428 (20.2)	289 (22.9)	
¥1001 to ¥3000	2309 (66.3)	73 (72.3)	1399 (65.9)	837 (66.3)	
≥¥3000	447 (12.8)	16 (15.8)	295 (13.9)	136 (10.8)	
Alcohol use, %					<0.001
Yes	474 (13.6)	40 (39.6)	380 (17.9)	54 (4.3)	
No	3013 (86.4)	61 (60.4)	1744 (82.1)	1208 (95.7)	
Waist-hip ratio	0.9 ± 0.1	0.9 ± 0.0	0.9 ± 0.1	0.9 ± 0.1	<0.001
TG, mmol/L	1.7 ± 1.5	3.2 ± 3.1	1.8 ± 1.5	1.4 ± 1.1	<0.001
LDL-C, mmol/L	2.6 ± 0.8	3.2 ± 1.0	2.7 ± 0.8	2.4 ± 0.6	<0.001
HDL-C, mmol/L	1.6 ± 0.5	1.6 ± 0.4	1.6 ± 0.4	1.7 ± 0.5	<0.001
UA, μmol/L	289.4 ± 89.6	337.4 ± 92.9	304.3 ± 90.4	260.4 ± 79.5	<0.001
Homocysteine, mol/L	15.2 ± 9.2	17.1 ± 9.2	16.4 ± 9.5	13.0 ± 8.1	<0.001
CRP, mg/dL	2.0 ± 3.9	2.9 ± 2.7	2.3 ± 4.4	1.5 ± 2.7	<0.001

Abbreviations: CVH, cardiovascular health; ¥, Yuan; TG, triglycerides; LDL-C, low-density lipoprotein cholesterol; HDL-C, high-density lipoprotein cholesterol; UA, uric acid; CRP, C-reactive protein.

The distributions of the CVH metrics are demonstrated in Table 2. The ideal smoking, BMI, physical activity and healthy diet status was presented in 64.55% (2,251/3,487), 53.40% (1,862/3,487), 39.03% (1,361/3,487), and 21.16% (738/3,487) of the participants, respectively. The ideal TC, BP and FBG status was presented in 58.88% (2,053/3,487), 19.73% (688/3,487) and 68.66% (2,394/3,487) of the participants, respectively. The different grades of each baseline CVH metric were significantly correlated with the occurrence of ECAS. The distribution of the inadequate, average, and optimum groups was 2.90% (101/3,487), 60.91% (2,124/3,487), and 36.19% (1,262/3,487), respectively. Both the sum and grade of the CVH score were negatively associated with the incidence of ECAS (Supplementary Fig. S1).

**Table 2:** Distribution of baseline cardiovascular health metrics between the participants who developed into ECAS or not in two-year follow-up.

	Total (n = 3487)	ECAS		*P-value*
		Yes (n = 976)	No (n = 2511)	
Smoking				<0.001
Ideal	2251 (64.6)	570 (58.4)	1681 (67.0)	
Intermediate	183 (5.2)	85(8.7)	98 (3.9)	
Poor	1053 (30.2)	321 (32. 9)	732 (29.2)	
BMI				0.045
Ideal	1862 (53.4)	493 (50.5)	1369 (54.5)	
Intermediate	1410 (40.4)	427 (43.8)	983 (39.2)	
Poor	215 (6.2)	56 (5.7)	159 (6.3)	
Physical exercise				<0.001
Ideal	1361 (39.0)	306 (31.4)	1055 (42.0)	
Intermediate	915 (26.2)	206 (21.1)	709 (28.2)	
Poor	1211 (34.7)	464 (47.5)	747 (29.8)	
Salt intake				0.011
Ideal	738 (21.2)	191 (19.6)	547 (21.8)	
Intermediate	2046 (58.7)	557 (57.1)	1489 (59.3)	
Poor	703 (20.2)	228 (23.4)	475 (18.9)	
TC				<0.001
Ideal	2053 (58.9)	455 (46.6)	1598 (63.6)	
Intermediate	1024 (29.4)	356 (36.5)	668 (26.6)	
Poor	410 (11.8)	165 (16.9)	245 (9.8)	
BP				<0.001
Ideal	688 (19.7)	121 (12.4)	567 (22.6)	
Intermediate	1139 (32.7)	365 (37.4)	774 (30.8)	
Poor	1660 (47.6)	490 (50.2)	1170 (46.6)	
FBG				<0.001
Ideal	2394 (68.7)	619 (63.4)	1775 (70.7)	
Intermediate	787 (22.6)	231 (23.7)	556 (22.1)	
Poor	306 (8.8)	126 (12.9)	180 (7.2)	
CVH score	8.7 ± 2.2	7.8 ± 2.0	9.0 ± 2.2	<0.001
0–4 points	101 (2.9)	41 (4.2)	60 (2.4)	<0.001
5–9 points	2124 (60.9)	720 (73.8)	1404 (55.9)	
10–14 points	1262 (36.2)	215 (22.0)	1047 (41.7)	

Abbreviations: ECAS, extracranial carotid artery stenosis; BMI, body mass index; TC, total cholesterol; BP, blood pressure; FBG, fasting plasma glucose; CVH, cardiovascular health.

Table 3 presented the risk of ECAS events according to the three categories of the overall CVH scores. Model 1 represented an unadjusted analysis with an increased risk for the average and optimum groups (hazard ratio (HR): 0.63, 95% confidence interval (CI): 0.45–0.86; HR: 0.32, 95% CI: 0.23–0.45, respectively). In the full adjusted model (Model 4), using the inadequate group as a reference, the HRs for the average and optimum groups were 0.73 (95% CI: 0.52–1.03) and 0.58 (95% CI: 0.40–0.85), respectively. Overall, compared with the inadequate group, a gradient relationship was observed between the HR for ECAS and the CVH scores, i.e., the incidence of ECAS was negatively correlated with the CVH metrics summary score.

**Table 3:** The hazard ratio together with 95% confidence interval of the incident ECAS according to the summary CVH score in northern Han Chinese.

	Inadequate (0~4 points)	Average (5~9 points)		Optimum (10~14 points)	
		HR (95% CI)	*P-value*	HR (95% CI)	*P-value*
Model 1	ref	0.63 (0.45, 0.86)	0.004	0.32 (0.23, 0.45)	<0.001
Model 2	ref	0.66 (0.48, 0.92)	0.013	0.49 (0.34, 0.69)	<0.001
Model 3	ref	0.69 (0.50, 0.97)	0.032	0.54 (0.38, 0.78)	0.001
Model 4	ref	0.73 (0.52, 1.03)	0.073	0.58 (0.40, 0.85)	0.005

Model 1: unadjusted.

Model 2: adjusted for age and sex.

Model 3: adjusted for age, sex, weight, education, income, alcohol use, and waist-hip ratio.

Model 4: adjusted for age, sex, weight, education, income, alcohol use, waist-hip ratio, TG, LDL-C, HDL-C, UA, homocysteine, and CRP.

Abbreviations: ECAS, extracranial carotid artery stenosis; CVH, cardiovascular health; HR: hazard ratio; CI: confidence interval; TG, triglycerides; LDL-C, low-density lipoprotein cholesterol; HDL-C, high-density lipoprotein cholesterol; UA, uric acid; CRP, C-reactive protein.

Further risk analyses of the association between the ECAS incidence and each CVH metric were shown in Table 4. After adjusting for sex, age, and the other six CVH metrics, we found that, compared to the ideal status group, the intermediate and poor status TC groups had HRs of 1.54 (95% CI: 1.33–1.78) and 1.64 (95% CI: 1.37–1.97), respectively. The adjusted HRs for those with an intermediate and poor FBG were 1.29 (95% CI: 1.11–1.51) and 1.25 (95% CI: 1.02–1.52), respectively. The adjusted HR for inactivity was significantly associated with the incidence of ECAS (HR: 1.26; 95% CI: 1.08–1.47). However, no significant correlation was found between smoking, BMI, salt intake, BP or intermediate physical exercise and the incidence of ECAS.

**Table 4:** Risk of ECAS according to each CVH metric at baseline.

	Crude model			Adjusted model		
	HR	95% CI	*P-value*	HR	95% CI	*P-value*
Smoking						
Ideal	ref			ref		
Intermediate	2.11	1.68–2.67	<0.001	1.15	0.90–1.47	0.266
Poor	1.14	0.99–1.30	0.070	0.96	0.82–1.13	0.619
BMI						
Ideal	ref			ref		
Intermediate	1.09	0.95–1.24	0.209	1.03	0.90–1.18	0.695
Poor	0.97	0.74–1.28	0.840	1.04	0.78–1.38	0.778
Physical exercise						
Ideal	ref					
Intermediate	0.94	0.78–1.12	0.530	0.97	0.81–1.17	0.777
Poor	1.68	1.45–1.95	<0.001	1.26	1.08–1.47	0.003
Salt intake						
Ideal	ref					
Intermediate	1.03	0.87–1.22	0.138	1.09	0.92–1.29	0.300
Poor	1.20	0.99–1.45	0.071	1.17	0.96–1.43	0.129
TC						
Ideal	ref					
Intermediate	1.73	1.50–1.99	<0.001	1.54	1.33–1.78	<0.001
Poor	1.91	1.60–2.29	<0.001	1.64	1.37–1.97	<0.001
BP						
Ideal	ref					
Intermediate	1.95	1.59–2.40	<0.001	1.14	0.92–1.42	0.223
Poor	1.80	1.48–2.20	<0.001	1.01	0.82–1.25	0.893
FBG						
Ideal	ref					
Intermediate	1.29	1.11–1.51	0.001	1.29	1.11–1.51	0.001
Poor	1.59	1.31–1.93	<0.001	1.25	1.02–1.52	0.028

Adjusted model for sex, age, and the other six CVH metrics (TC, BP, FBG, smoking, BMI, physical activity, and salt intake).

Abbreviations: ECAS, extracranial carotid artery stenosis; CVH, cardiovascular health; HR: hazard ratio; CI: confidence interval; BMI, body mass index; TC, total cholesterol; BP, blood pressure; FBG, fasting plasma glucose.

## Discussion

In this two-year follow-up cohort study, 3,487 participants were included. In total, 976 participants were identified to experience new events of ECAS, and the incidence was 27.99% (976/3,487). The current study evaluated the longitudinal effects of an ideal baseline CVH status on the incidence of ECAS and showed that the optimum CVH status could decrease the risk of the incidence of ECAS by 42%. To the best of our knowledge, this study is the first attempt to demonstrate the relationship between CVH metrics and the incidence of ECAS in a longitudinal study.

Our findings indicate that CVH metrics are independent protective factors for the occurrence of ECAS (HR: 0.58; 95% CI: 0.40–0.85). ECAS is a significant upstream risk factor for stroke, and interventions targeting ECAS are important for the prevention of stroke^17^. However, the relationship of CVH metrics and ECAS has been relatively understudied. This study extended findings from previous studies focusing on the association between CVH metrics and the incidence of CVD events^18–20^ to the general population with ECAS. In another cross-sectional Asymptomatic Polyvascular Abnormalities Community (APAC) study, Zhang *et al*. reported a clear gradient relationship between the ideal CVH metrics and a lower prevalence of asymptomatic intracranial artery stenosis (ICAS)^21^. This study expanded the results obtained for ICAS to ECAS. Similarly, in the Mediators of Atherosclerosis in South Asians Living in America (MASALA) study, Talegawkar *et al*. found that the ideal CVH metrics are positively associated with lower levels of subclinical atherosclerosis as determined by coronary artery calcium and carotid intima-media thickness^22^. However, in the Age, Gene/Environment Susceptibility (AGES)-Reykjavik study, Sturlaugsdottir *et al*. reported that the CVH score is negatively associated with the total plaque area in older men, but not older women, but was not correlated with the progression of the total plaque area or carotid intima-media thickness over 5 years in either males or females^23^. Consequently, the association between CVH metrics and subclinical atherosclerosis should be further investigated in a large sample cohort study.

The relationship between the components of the CVH metrics and ECAS has been widely investigated in various populations. In the United States, Berger *et al*. investigated the association among 5 modifiable risk factors and carotid artery stenosis (≥50%) in 3,319,993 participants (aged 40 to 99) in the Life Line Screening Program between 2004 and 2008 and demonstrated that the adjusted odds ratios (ORs) for active smoking, sedentary lifestyle, hypercholesterolemia, hypertension, and diabetes are 1.77 (1.75–1.80), 1.17 (1.16–1.18), 1.45 (1.43–1.46), 1.62 (1.59–1.64) and 1.60 (1.57–1.63), respectively^24^. In the same population, Stein *et al*. reported that physical activity has a significantly lower OR of ECAS (OR: 0.80; 95% CI: 0.79–0.81) and that both physical activity intensity and frequency are associated with lower carotid artery stenosis in a graded manner (*P* trend < 0.0001)^25^. In India, Kaul *et al*. evaluated 1,500 asymptomatic individuals (>40 years) and found that smoking (OR: 3.6; 95% CI: 2.18–6.03), dyslipidemia (OR: 4.0; 95% CI: 2.52–6.63), hypertension (OR: 1.8; 95% CI: 1.11–2.96) and diabetes (OR: 2.3; 95% CI: 1.45–3.89) are significantly associated with carotid artery stenosis (>50%), particularly in those with more than 15 year of hypertension (OR: 2.5; 95% CI: 1.33–6.43), diabetes (OR: 6.2; 95% CI: 3.41–11.3), and smoking (OR: 5.2; 95% CI: 2.20–12.1)^26^. Nevertheless, inconsistent data remain. Among 96 patients with asymptomatic ECAS (≥50%), Ehrhardt *et al*. found that smoking, BMI, dyslipidemia and diabetes has not predictive value for the ECAS severity (OR: 1.06, 95% CI: 0.25–4.44, *P* = 0.94; OR: 0.92, 95% CI: 0.80–1.05, *P* = 0.20; OR: 0.98, 95% CI: 0.29–3.31, *P* = 0.98; OR: 2.61, 95% CI: 0.46–14.98, *P* = 0.28, respectively)^27^. Song *et al*. also showed that smoking, BMI, dyslipidemia and hypertension are not associated with the maximum carotid intima-media thickness in 252 Korean patients with acute ischemic stroke (*P* = 0.021, 0.063, 0.319, 0.559 for men; *P* = 0.072, 0.586, 0.184, 0.934 for women)^28^. Nonetheless, these observations were limited by the design of the cross-sectional study. Our observation that ideal physical activity, TC and FBG are protective factors against ECAS is consistent with most studies. In addition, the association between the CVH score and smoking, BMI, salt intake and BP are not significant, which could be due to the ethnic difference or insufficient sample size.

The current study reinforces pursuing modifiable risk factors, including lifestyle and drug use, to prevent stroke. The components of the CVH metrics are considered to be modifiable risk factors; thus, improvement in the CVH status may potentially reduce ECAS or subsequent stroke morbidity. Considering the poor risk factor control and low prevalence of the optimum category (36.19%, 1,262/3,487) revealed in our study, improvement is needed to reduce the risks of ECAS events. For example, actively participating in physical exercise is a clearly attainable objective. These findings also provide clues for the debated screening strategy for asymptomatic ECAS in the general population^3^, supporting that the potential involvement of ideal physical activity, TC and FBG in the screening strategy had been used to identify high-risk groups.

Several limitations in this study should be considered. First, compared with the baseline characteristics of the study subjects, those of the lost subjects were an elderly age, mainly male gender, lower education level, higher waist-hip ratio, higher FBG, higher BP, higher homocysteine, and worse CVH status, which increase the susceptibility to atherosclerosis^6,29^. Therefore, the failure to include certain follow-up subjects might lead to the underestimation of the risk of the CVH metrics on the occurrence of ECAS in our study. Second, the current results were obtained from a community cohort in northern China, which may restrict the further generalization. Third, the measures of the CVH metrics might result in bias to a certain degree. Salt intake is used as a proxy for a modified definition of diet, and the data regarding the smoking status, physical activity status and dietary intake were obtained from self-reported measures.

## Conclusions

Our study demonstrated that a poor baseline CVH status was associated with the occurrence of ECAS during the follow-up period. This study provides practical insight for further developing effective screening strategies or implementing the best medical treatment.

## Methods

### Study design and participants

The design, methods and baseline characteristics of the APAC study have been described previously^30^. The APAC study is a prospective population study based on the Kailuan community in Tangshan City, which is a large industrial city located in North China. In the present study, the inclusion criteria were as follows: (1) aged 40 years or older and (2) complete data of CVH metrics. We excluded participants with a history of transient ischemic attack, stroke, and coronary heart disease. Participants who had ECAS from June 2010 to June 2011 were also excluded. All participants provided informed consents. The subjects received follow-up evaluations in 2012–2013 were included in the final analysis.

**Figure 1:**
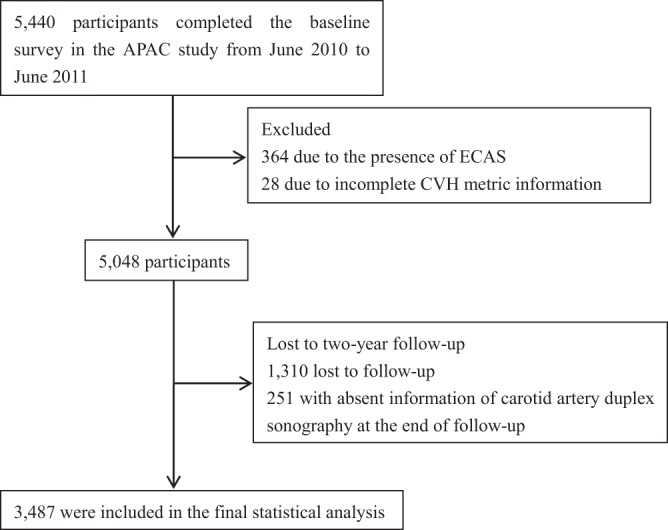
Flow chart of the study to show the selection of APAC study participants for analysis. Abbreviations: APAC, Asymptomatic Polyvascular Abnormalities Community; ECAS, extracranial carotid artery stenosis; CVH, cardiovascular health.

The APAC study involved 5,440 participants. In total, 364 participants were excluded because of the presence of ECAS during the baseline examination, 28 participants were excluded because of missing information regarding any of the CVH metrics, and finally, 5,048 participants were included in the cohort. After a 2-year follow-up, 3,738 subjects participated in the follow-up visit, 251 participants failed to undergo carotid ultrasound examinations, and finally, 3,487 participants with complete information in both surveys were included in this study (Fig. 1).

The mean follow-up period was 2 years, yielding a total of 7,968.59 person-years follow-up. Compared with the study population, the baseline characteristics of the lost group were worse and included an elderly age, the male sex, lower education level, higher waist-hip ratio, higher FBG, higher BP, higher homocysteine, and lower CVH score category (Table 5).

**Table 5:** Comparisons of baseline data between the participants included in the final analysis and these lost to follow-up.

	Participated in study (n = 3487)	Lost to follow-up (n = 1561)	*P-value*
Age, years	53.9 ± 10.7	56.7 ± 12.7	<0.001
Sex, %			
Female	1554 (44.6)	557 (35.7)	<0.001
Male	1933 (55.4)	1004 (64.3)	
Body weight, kg	69.0 ± 11.0	69.5 ± 11.0	0.127
Education level, %			
Illiterate/primary school	343 (9.8)	254 (16.3)	<0.001
Middle school	1483 (42.5)	752 (48.2)	
High school or above	1660 (47.6)	555 (35.6)	
Income, ¥/month, %			
<¥1000	729 (21.0)	354 (22.7)	0.693
¥1001 to ¥3000	2309 (66.3)	1039 (66.6)	
≥¥3000	447 (12.8)	168 (10.8)	
Smoking, %			
Yes	1053 (30.2)	505 (32.4)	0.126
No	2434 (69.8)	1056 (67.7)	
Alcohol use, %			
Yes	474 (13.6)	239 (15.3)	0.105
No	3013 (86.4)	1322 (84.7)	
Waist-hip ratio	0.9 ± 0.1	0.9 ± 0.1	<0.001
BMI, kg/m^2^	25.0 ± 3.2	25.1 ± 3.4	0.144
TC, mmol/L	5.1 ± 1.0	5.1 ± 1.0	0.456
TG, mmol/L	1.7 ± 1.5	1.7 ± 1.3	0.183
LDL-C, mmol/L	2.6 ± 0. 8	2.6 ± 0.8	0.624
HDL-C, mmol/L	1.6 ± 0.5	1.6 ± 0.4	0.115
FBG, mg/dL	99.5 ± 26.1	102.3 ± 29.1	0.001
SBP, mmHg	129.5 ± 19.4	134.1 ± 20.5	<0.001
DBP, mmHg	82.7 ± 11.0	83.7 ± 11.2	0.004
UA, μmol/L	289.4 ± 89.6	285.4 ± 90.2	0.150
Homocysteine, μmol/L	15.2 ± 9.2	16.8 ± 10.3	<0.001
CRP, mg/dL	1.0 (0.5, 2.1)	1.0 (0.6, 2.2)	0.003
CVH score			
0–4 points	101 (2.9)	50 (3.2)	0.001
5–9 points	2124 (60.9)	1031 (66.1)	
10–14 points	1262 (36.2)	480 (30.8)	

Abbreviations: ¥, Yuan; BMI, body mass index; TC, total cholesterol; TG, triglycerides; LDL-C, low-density lipoprotein cholesterol; HDL-C, high-density lipoprotein cholesterol; FBG, fasting plasma glucose; SBP, systolic blood pressure; DBP, diastolic blood pressure; UA, uric acid; CRP, C-reactive protein; CVH, cardiovascular health.

### Ethics statement

The study was performed according to the guidelines of the Declaration of Helsinki and was approved by the Ethics Committee of the Kailuan General Hospital. Written informed consent was obtained from each participant.

### Assessment of cardiovascular health metrics and potential covariates

Information was collected regarding the following variables: age, gender, weight, education level, average income of each family member, medical history, family medical history, alcohol consumption, height, BMI, waist circumference, hip circumference, waist-hip ratio, smoking status, physical exercise, dietary data, BP, FBG, TC, TG, LDL-C, HDL-C, UA, homocysteine, and CRP. The data collection method has been previously described^30^.

CVH metrics consisting of 4 health behaviors (smoking, BMI, physical exercise, and diet) and 3 health factors (TC, BP, and FBG) were measured using standard procedures as previously described^21^ (Supplementary Table S1). Excessive salt intake is frequently observed among the majority of the Chinese population and is closely associated with the risk of stroke events^31^; therefore, daily salt intake has been used as an alternative measure for the dietary metrics in several studies^32,33^. Each metric was defined as poor (0 point), intermediate (1 point) or ideal (2 points) by Huffman *et al*.^34^. All participants were grouped into the following three categories according to the sum of the scores on the 7 CVH metrics: inadequate (0~4 points), average (5~9 points), and optimum (10~14 points)^18,35^.

### Follow-up and outcome assessment

A face-to-face interview was adopted for this cohort at the follow-up visit up to December 31, 2013. The physicians and nurses who were involved in this follow-up were blinded to the baseline data.

The main outcome was the occurrence of ECAS during the two-year follow-up. ECAS refers to the presence of atherosclerotic narrowing of the common carotid artery, carotid bifurcation, or extracranial internal carotid^3^. Duplex sonography is a widely used, noninvasive, easily performed, and cost-effective initial diagnostic imaging method with high sensitivity and specificity in ECAS evaluation^4,6,36^.

The method used to evaluate ECAS has been previously described^17,30,37^. In brief, all participants underwent a bilateral carotid duplex sonography. ECAS was defined as a peak systolic blood flow velocity ≥125 cm/s and a vertical artery peak systolic blood flow velocity ≥170 cm/s in the common carotid artery or internal carotid artery. Stenosis was graded according to the diagnostic criteria of the Society of Radiologists in the Ultrasound Consensus Conference in 2003^38^. In our study, the degree of stenosis was classified as none, <50% (mild), andv ≥50% (moderate to severe) involving the bilateral internal or common carotid artery. When both carotid arteries were measured, the most severe grade was recorded as the outcome.

### Statistical analyses

The normally distributed continuous variables are presented as the mean ± SD and were compared using a Student’s *t*-test or analysis of variance (ANOVA). For continuous variables with a non-normal distribution and graded variables, the data are presented as the median (interquartile range) and were compared using the Wilcoxon rank sum tests. The categorical variables are presented as a number (percentage) and were compared using chi-square tests.

Cox proportional hazards models were used to assess the ECAS events risk by calculating the HR and 95% CI. The confounding factors were adjusted. For the overall CVH score, the lowest category was used as the reference group, the Cox proportional hazards model used a univariate model, and multivariate modeling was performed to evaluate the impact of the overall CVH metric scores on the incidence of ECAS. ECAS events were the dependent variables, while the categories of the CVH scores were the independent variables. The multivariate Cox proportional hazards model was performed separately for the seven metrics. Using the ideal status as a reference for each metric, the HR and 95% CI for the risk of ECAS events were calculated after adjusting for sex, age, and the other six metrics.

The statistical analyses were performed using SAS software version 9.3 (SAS Institute, Cary, NC). All statistical tests were 2-sided, and the statistically significant level was set at *P* < 0.05.

### Data availability statement

All relevant data are published in the paper and its supporting additional files.

## Electronic supplementary material

Supplementary Information

## References

[1] Mozaffarian D (2015). Heart disease and stroke statistics–2015 update: a report from the American Heart Association. Circulation.

[2] Lozano R (2012). Global and regional mortality from 235 causes of death for 20 age groups in 1990 and 2010: a systematic analysis for the Global Burden of Disease Study 2010. Lancet.

[3] Jonas DE (2014). Screening for asymptomatic carotid artery stenosis: a systematic review and meta-analysis for the U.S. Preventive Services Task Force. Ann Intern Med.

[4] Eckstein HH (2013). The diagnosis, treatment and follow-up of extracranial carotid stenosis. Dtsch Arztebl Int.

[5] Kirshner HS (2009). Differentiating ischemic stroke subtypes: risk factors and secondary prevention. J Neurol Sci.

[6] Huynh TT, Broadbent KC, Jacob AD, James S, Erasmus JJ (2015). Screening for carotid artery stenosis. Semin Roentgeno.

[7] Shah PK (2010). Screening asymptomatic subjects for subclinical atherosclerosis: can we, does it matter, and should we. J Am Coll Cardiol.

[8] Lloyd-Jones DM (2010). Defining and setting national goals for cardiovascular health promotion and disease reduction: the American Heart Association’s strategic Impact Goal through 2020 and beyond. Circulation.

[9] Bambs C, Reis SE (2011). Embracing primordial prevention for ideal cardiovascular health. Future Cardio.

[10] Fang N, Jiang M, Fan Y (2016). Ideal cardiovascular health metrics and risk of cardiovascular disease or mortality: A meta-analysis. Int J Cardiol.

[11] Maclagan LC, Tu JV (2015). Using the concept of ideal cardiovascular health to measure population health: a review. Curr Opin Cardiol.

[12] Dong C (2012). Ideal cardiovascular health predicts lower risks of myocardial infarction, stroke, and vascular death across whites, blacks, and hispanics: the northern Manhattan study. Circulation.

[13] Yan N (2016). Association of ideal cardiovascular health and brachial-ankle pulse wave velocity: a cross-sectional study in northern China. J Stroke Cerebrovasc Dis.

[14] Qiu J (2016). The association between ankle-brachial index and asymptomatic cranial-carotid stenosis: a population-based, cross-sectional study of 5440 Han Chinese. Eur J Neurol.

[15] Wang Y (2017). Association between ideal cardiovascular health metrics and suboptimal health status in Chinese population. Sci Rep.

[16] Hao Z (2016). The Association between Ideal Cardiovascular Health Metrics and Extracranial Carotid Artery Stenosis in a Northern Chinese Population: A Cross-Sectional Study. Sci Rep.

[17] Wang D (2016). Asymptomatic Extracranial Artery Stenosis and the Risk of Cardiovascular and Cerebrovascular Diseases. Sci Rep.

[18] Kulshreshtha A (2013). Life’s Simple 7 and risk of incident stroke: the reasons for geographic and racial differences in stroke study. Stroke.

[19] Lachman S (2016). Ideal cardiovascular health and risk of cardiovascular events in the EPIC-Norfolk prospective population study. Eur J Prev Cardiol.

[20] Ford ES, Greenlund KJ, Hong Y (2012). Ideal cardiovascular health and mortality from all causes and diseases of the circulatory system among adults in the United States. Circulation.

[21] Zhang Q (2013). Ideal cardiovascular health metrics on the prevalence of asymptomatic intracranial artery stenosis: a cross-sectional study. PLoS One.

[22] Talegawkar SA, Jin Y, Kandula NR, Kanaya AM (2017). Cardiovascular health metrics among South Asian adults in the United States: Prevalence and associations with subclinical atherosclerosis. Prev Med.

[23] Sturlaugsdottir R (2015). Carotid atherosclerosis and cardiovascular health metrics in old subjects from the AGES-Reykjavik study. Atherosclerosis.

[24] Berger JS (2013). Modifiable risk factor burden and the prevalence of peripheral artery disease in different vascular territories. J Vasc Surg.

[25] Stein RA (2015). Association between physical activity and peripheral artery disease and carotid artery stenosis in a self-referred population of 3 million adults. Arterioscler Thromb Vasc Biol.

[26] Kaul S (2017). Prevalence and risk factors of asymptomatic carotid artery stenosis in Indian population: An 8-year follow-up study. Neurol India.

[27] Ehrhardt J (2015). Sleep apnea and asymptomatic carotid stenosis: a complex interaction. Chest.

[28] Hyun S (2015). Can the Neutrophil-to-Lymphocyte Ratio Appropriately Predict Carotid Artery Stenosis in Patients with Ischemic Stroke?-A Retrospective Study. J Stroke Cerebrovasc Dis.

[29] Paraskevas KI, Mikhailidis DP, Veith FJ, Spence JD (2016). Definition of Best Medical Treatment in Asymptomatic and Symptomatic Carotid Artery Stenosis. Angiology.

[30] Zhou Y (2013). Asymptomatic polyvascular abnormalities in community (APAC) study in China: objectives, design and baseline characteristics. PLoS One.

[31] Shao S (2017). Salt reduction in China: a state-of-the-art review. Risk Manag Healthc Policy.

[32] Wu S (2012). Prevalence of ideal cardiovascular health and its relationship with the 4-year cardiovascular events in a northern Chinese industrial city. Circ Cardiovasc Qual Outcomes.

[33] Zhang Q (2013). Ideal cardiovascular health metrics and the risks of ischemic and intracerebral hemorrhagic stroke. Stroke.

[34] Huffman MD (2012). Cardiovascular health behavior and health factor changes (1988–2008) and projections to 2020: results from the National Health and Nutrition Examination Surveys. Circulation.

[35] Miao C (2015). Cardiovascular Health Score and the Risk of Cardiovascular Diseases. PLoS One.

[36] Pandya A, Gupta A (2016). Improving imaging to optimize screening strategies for carotid artery stenosis. Clin Imaging.

[37] Wang J (2014). Elevated fasting glucose as a potential predictor for asymptomatic cerebral artery stenosis: a cross-sectional study in Chinese adults. Atherosclerosis.

[38] Grant EG (2003). Carotid artery stenosis: gray-scale and Doppler US diagnosis–Society of Radiologists in Ultrasound Consensus Conference. Radiology.

