# Supplementary material for: Ideal Cardiovascular Health Metrics Associated with Reductions in the Risk of Extracranial Carotid Artery Stenosis: a Population-based Cohort Study
Source: Sci Rep. 2018 Aug 16;8:12277. doi: 10.1038/s41598-018-29754-3 (PMC6095842; doi:10.1038/s41598-018-29754-3)
Supplement: Supplementary file 1 — Supplementary Information [file 41598_2018_29754_MOESM1_ESM.pdf]

Title: Ideal cardiovascular health metrics associated with reductions in the risk of extracranial carotid artery stenosis: a population-based cohort study

Junyou Wang[1]*, Bo Shao[1]*, Da Lin[1], Xijun He[1], Yongqiang Zhang[2], Li Zhang[2], Tian Jiang[3], Jinzhong Xu[4], Youxin Wang[5], Jing Wu[6], Yong Zhou[7], Junzheng Chen[8] & Lingfang Teng[1]

1 Department of Neurosurgery, The First People’s Hospital of Wenling, Wenling, 317500, China.

2 Department of Neurology, The First People’s Hospital of Wenling, Wenling, 317500,China.

3 Department of Central Lab, The First People’s Hospital of Wenling, Wenling, 317500, China.

4 Department of Clinical Pharmacy, The First People’s Hospital of Wenling, Wenling, 317500, China.

5 Beijing Key Laboratory of Clinical Epidemiology, School of Public Health, Capital Medical University, Beijing 100069, China.

6 Beijing Recdata Technology Co., Ltd., Beijing, 100050, China.

7 Department of Cardiology, Beijing An Zhen Hospital, Capital Medical University. Beijing Institute of Heart, Lung and Blood Vascular Diseases, Beijing, 100029, China.

8 Department of General Surgery, The First People’s Hospital of Wenling, Wenling, 317500, China.

* Junyou Wang and Bo Shao contributed equally to this study.

Correspondence and requests for materials should be addressed to J.C. (e-mail: wlsphchen@163.com) or L.T. (e-mail: wangjammy@126.com).

SUPPLEMENTARY

Supplementary Table S1. Definition of CVH metrics used in this study.

Measures	Definition
Smoking	
Ideal	never or quit smoking >12 months
Intermediate	former smoking ≤12 months
Poor	currently smoking
BMI	
Ideal	<25 kg/m^2
Intermediate	25–29.9 kg/m^2
Poor	≥30 kg/m^2
Physical exercise	
Ideal	≥150 min/week of moderate intensity or ≥75 min/week of vigorous intensity
Intermediate	1–149 min/week of moderate intensity or 1–74 min/week of vigorous intensity
Poor	0 minutes of moderate or vigorous exercise per week
Salt	
Ideal	low salt intake (<6 g per day)
Intermediate	intermediate salt intake (6–10 g per day)
Poor	high salt intake (>10 g per day)
TC	
Ideal	<200 mg/dL in the untreated population
Intermediate	200–239 mg/dL in the untreated population or population treated with lipid-lowering medicines with ideal TC levels
Poor	≥240 mg/dL in the untreated population or population treated with lipid-lowering medicines with intermediate TC levels
BP	
Ideal	SBP<120 mmHg and DBP<80 mmHg in the untreated population
Intermediate	120 mmHg≤SBP≤139 mmHg or 80 mmHg≤DBP≤89 mmHg in the untreated population or population treated with antihypertensive agents with ideal BP levels
Poor	SBP≥140 mmHg or DBP≥90 mmHg in the untreated population or population treated with antihypertensive agents with intermediate BP levels
FBG	
Ideal	<100 mg/dL in the untreated population
Intermediate	100–125 mg/dL in the untreated population or population treated with hypoglycemic agents with an ideal FBG level
Poor	≥126 mg/dL in the untreated population or population treated with hypoglycemic agents with intermediate FBG levels

Abbreviations: CVH, cardiovascular health; BMI, body mass index; TC, total cholesterol; BP, blood pressure; SBP, systolic blood pressure; DBP, diastolic blood pressure; FBG, fasting plasma glucose.

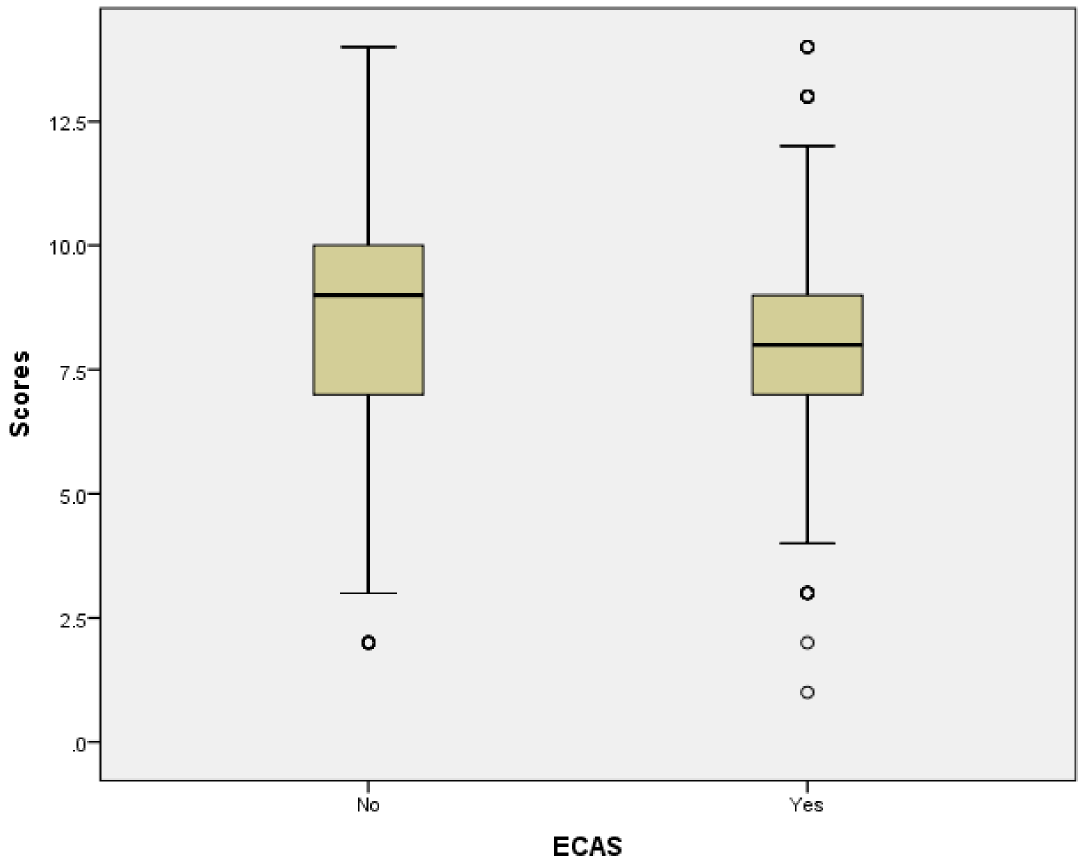

Supplementary Figure S1. The relationship between the CVH score and the quantitative values of ECAS. The improvement of the CVH score would reduce the risk of developing ECAS. Abbreviations: CVH, cardiovascular health; ECAS, extracranial carotid artery stenosis.
